# P-973. Pediatric Infectious Diseases rotation for general pediatric residents in Washington DC: impact, benefits and pitfalls

**DOI:** 10.1093/ofid/ofae631.1163

**Published:** 2025-01-29

**Authors:** Mohamad Shieb, Eric J Stern, Susana Gaviria, Badr AlSayeb, Elizabeth H Ristagno, Guyu Li

**Affiliations:** medstar georgetown university hospital, ARLINGTON, Virginia; Medstar Georgetown Pediatrics, Washington, District of Columbia; Medstar Georgetown University Hospital, District of Columbia, District of Columbia; Medstar Georgetown University Hospital, District of Columbia, District of Columbia; Mayo Clinic, Rochester, MN; Mayo Clinic, Rochester, MN

## Abstract

**Background:**

Although infectious diseases present significant challenges in pediatric care, formal training in pediatric infectious diseases is not mandated in categorical pediatric residency training. This research seeks to examine the self-assessed confidence levels of general pediatric residents in Washington DC in managing common infectious diseases. The study specifically compares the confidence levels of residents who have undergone elective infectious diseases training with those who have not.

Subjective Confidence in the Management of Common Infectious Diseases Encounters

**Figure:**
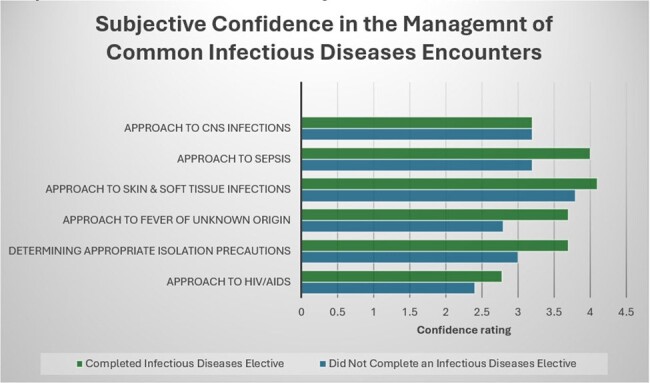

**Methods:**

A cross-sectional study was conducted using voluntary surveys distributed among pediatric residents from various programs in Washington DC. The surveys aimed to assess subjective confidence levels in managing common infectious disease conditions and to explore motivation to pursue infectious disease training.

Effect of infectious diseases elective rotation on interest in infectious diseases as a career.

**Figure:**
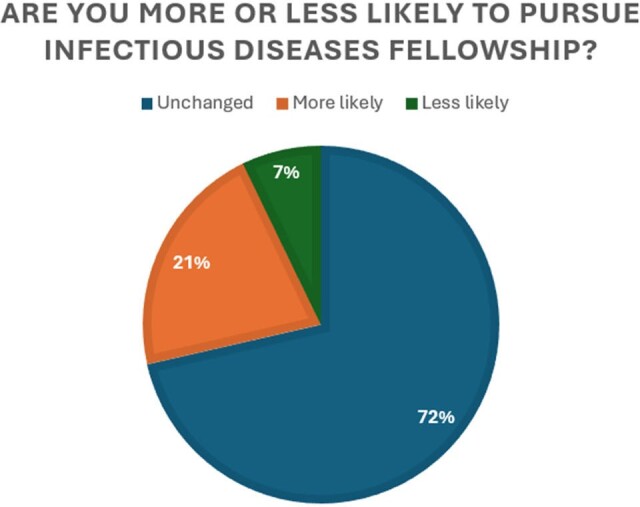

Are you more or less likely to pursue infectious diseases fellowship?

**Results:**

Analysis of 20 completed surveys revealed that residents who completed infectious diseases rotations demonstrated significantly higher confidence levels in managing infectious disease conditions compared to their counterparts (P: < 0.001). Notably, residents with specialized training showed increased confidence in antibiotic selection and in their diagnostic abilities. Additionally, they exhibited enhanced confidence in managing specific presentations such as sexually transmitted diseases, fever of unknown origin, and central nervous system infections (all with P < 0.05). Finally, 22% of respondents expressed an increased likelihood of pursuing a career in infectious diseases following their rotation.

Effect of infectious diseases rotation on the confidence in the management of inpatient and outpatient infectious diseases encounters

**Figure:**
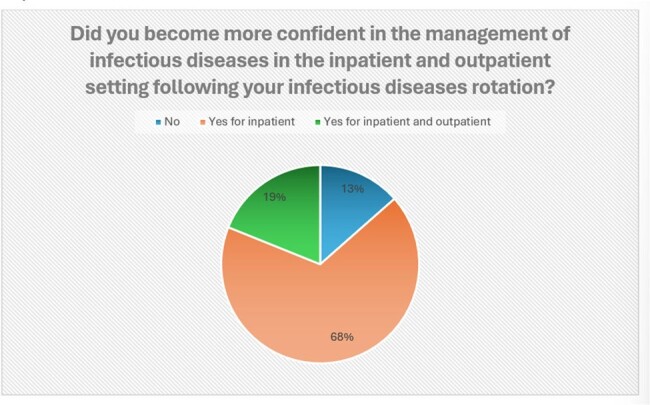

**Conclusion:**

These findings underscore the potential benefits of incorporating infectious diseases rotations into pediatric residency programs, as evidenced by improved skills in antibiotic selection, diagnosis, and management of specific infectious disease scenarios. Additionally, the observed increased interest in pursuing a career in infectious diseases among a subset of residents highlights the positive impact of such training on career trajectory and specialty choice. Investigators plan to distribute the questionnaire more broadly across the US to see if these results are more widespread.

**Disclosures:**

**All Authors**: No reported disclosures

